# Observations on the Effects of Magistery of Bismuth, Given Internally, as an Antispasmodic

**Published:** 1786

**Authors:** Lewis Odier

**Affiliations:** Physician at Geneva, Honorary Member, and formerly one of the annual Presidents, of the Medical Society of Edinburgh


XIII. *Observations on the Effects of Magistery of Bismuth, given internally, as an Antispasmodic. By* Lewis Odier, *M. D. Physician at Geneva, Honorary Member, and formerly one of the annual Presidents, of the Medical Society of Edinburgh.*—Vide *Journal de Medecine. Chirurgie, Pharmacie,* &c. Tome LXVII. 8vo. Paris, 1786.

WE have here a farther account of the new remedy mentioned in our last volume *. The disease in which this medicine is said to be particularly efficacious is a painful affection, or cramp, as Dr. Odier calls it, of the stomach, which comes on after meals, and which he supposes to be owing to a morbid irritability of that organ. In one instance of a complaint of this sort, which had continued

* Vol. VI. page 406.

during fifteen years, and for which a variety of ſedative and ſtomachic medicines had been adminiſtered without effect, a permanent cure, he aſſures us, was accompliſhed, in the ſpace of a few days, by the uſe of the magiſtery of biſmuth.

In other nervous affections, which have ſeemed to depend on the general irritability of the ſyſtem, rather than on any peculiar exceſs of irritability of the ſtomach, as, for example, in hyſterical and epileptic caſes, Dr. Odier has ſeldom, he tells us, ſeen it ſucceed. Even in complaints of this ſort, however, he has now and then, he aſſures us, experienced the beſt effects from it, eſpecially when the paroxyſms have been extremely violent. He has likewiſe, he adds, in ſeveral inſtances, found it of uſe in removing palpitations, pains of the ſtomach, and other nervous affections in pregnant women.

Dr. Odier directs this medicine to be given in powder, mixed with a little pectoral ſyrup and water. The doſe is from two to twelve grains four times a day, a quarter of an hour before each meal.

In general, he aſſures us, it produces no ſenſible effect; but in ſome few caſes he has

ſeen

ſeen it excite diſagreeable effects, ſuch as nauſea, vomiting, diarrhœa, a ſenſation of heat in the cheſt, and giddineſs. Theſe effects, however, he thinks, depended on ſome peculiarity of temperament in the patients, rather than on the doſes in which the magiſtery was adminiſtered, as in thoſe particular caſes very ſmall doſes uniformly produced the ſame effects as when much larger doſes were given ; and oftentimes ſmall doſes, he obſerves, have appeared in ſome reſpects to diſagree with patients, who have afterwards been very well able to ſupport much larger ones ; ſo that as yet he knows not the limits of the remedy, or, in other words, the doſes beyond which it cannot be extended without inconvenience.

Dr. Odier has ſeen ſeveral patients, who, at firſt, experienced no relief from the medicine, but who afterwards, in proportion as the doſe was augmented, found it gradually more and more efficacious till they were cured. On the other hand, he has met with patients, who, from a very ſmall doſe, have immediately received from it either a complete cure, or all the relief the medicine was capable of affording them, and who gained nothing by taking it in larger doſes, although they were able to ſupport them without the leaſt inconvenience.

Of ſeventy-eight patients to whom our author has adminiſtered this medicine, thirty-ſix, he tells us, have been perfectly cured by it. The greater number of theſe, it ſeems, were ſubject to the painful affection of the ſtomach, (or *dyſpepſia*, as we would call it) in which he finds the magiſtery of biſmuth particularly ſucceſsful. Of the forty two other patients, ſeventeen were more or leſs relieved by it; fourteen omitted to inform him of the effects of the medicine; and the remaining eleven found it of no uſe. The greater part of theſe laſt, he obſerves, were perſons in an infirm ſtate of health, who had long laboured under diſeaſes of a complicated and incurable nature.

To what Dr. Odier has ſaid on this ſubject we can add but little from our own experience; for although we have had recourſe to the magiſtery of biſmuth in five or ſix caſes of dyſpepſia, without ſeeing it produce any good effect, yet, as we have never extended the doſe of it beyond four grains, and have given it only to ſo ſmall a number of patients, we are far from conſidering our trials with it as concluſive, eſpecially as in all of thoſe caſes the dyſpeptic

ſymptoms

ſymptoms were apparently complicated with marks of general debility, and had reſiſted a variety of remedies. In two of the patients it occaſioned nauſea and head-ach, in doſes of one or two grains; but theſe effects immediately ceaſed upon laying aſide the medicine.

